# Investigation of amino acid specificity in the CydX small protein shows sequence plasticity at the functional level

**DOI:** 10.1371/journal.pone.0198699

**Published:** 2018-06-18

**Authors:** Jessica J. Hobson, Austin S. Gallegos, Benjamin W. Atha, John P. Kelly, Christina D. Lein, Cailtin E. VanOrsdel, John E. Weldon, Matthew R. Hemm

**Affiliations:** Department of Biological Sciences, Towson University, Towson, MD, United States of America; Russian Academy of Medical Sciences, RUSSIAN FEDERATION

## Abstract

Small proteins are a new and expanding area of research. Many characterized small proteins are composed of a single hydrophobic α-helix, and the functional requirements of their limited amino acid sequence are not well understood. One hydrophobic small protein, CydX, has been shown to be a component of the cytochrome *bd* oxidase complex in *Escherichia coli*, and is required for enzyme function. To investigate small protein sequence specificity, an alanine scanning mutagenesis on the small protein CydX was conducted using mutant alleles expressed from the *E*. *coli* chromosome at the wild-type locus. The resulting mutant strains were assayed for CydX function. No single amino acid was required to maintain wild-type resistance to β-mercaptoethanol. However, substitutions of 10-amino acid blocks indicated that the N-terminus of the protein was required for wild-type CydX activity. A series of double mutants showed that multiple mutations at the N-terminus led to β-mercaptoethanol sensitivity *in vivo*. Triple mutants showed both *in vivo* and *in vitro* phenotypes. Together, these data provide evidence suggesting a high level of functional plasticity in CydX, in which multiple amino acids may work cooperatively to facilitate CydX function.

## Introduction

Small proteins, defined here as those containing 75 or fewer amino acids, are a poorly understood fraction of the proteome. Their small size makes them challenging to identify using standard biochemical methods, and it is difficult to identify true short genes out of the thousands of short open reading frames (sORFs) contained in any genome [1–3]. In addition, characterizing of the function of small proteins (SPs) is often more challenging due to experimental factors related to their small size [4]. For example, it may be difficult to develop antibodies that recognize a 37-amino acid hydrophobic SP, yet adding a 10–30 amino acid epitope tag may substantially affect the protein’s function. Finally, given the small size of sORFs, they may be missed in genetic screens identifying mutations that lead to particular phenotypes [5,6]. Altogether, these technical constraints mean that, compared to larger proteins, relatively little is known about small protein abundance, function, and biochemical characteristics.

Despite these challenges, it is increasingly clear that SPs play important roles in cell biology [3, 7]. In prokaryotes, SPs have been found to recognize membrane curvature during spore formation, activate a multi-drug efflux pump, and serve as components of multi-protein complexes [8–11]. In eukaryotes, short genes have been discovered on RNAs that were previously thought to be long, non-coding RNAs, and the SPs they encode have been found to play essential roles in *Drosophila* leg development, *Tribolium* development and cardiac contraction [12–14]. Many identified small proteins, including MgtR, AcrZ, and CydX in *E*. *coli*, as well as the O3 protein in the vaccinia virus, are predicted to consist of a single hydrophobic α-helix and localize to the cell membrane [15, 9, 10]. Considering the growing evidence that there are many different small hydrophobic, α-helical proteins at the cell membrane, it remains a uncertain how these small transmembrane proteins achieve specificity of binding and function with their protein partners and not with other membrane complexes.

Although few mutagenesis studies of SPs have been conducted, SPs that have been characterized show intriguing differences in sequence specificity. Work with the *E*. *coli* MgtR protein has shown that it has individual residues that are essential for protein function [15]. This small protein regulates the levels of the magnesium transporter, MgtC, by binding to it and recruiting the AAA+ protease FtsH for MgtC degradation. Single mutations of two residues out of 11 tested eliminated protein activity and abolished the interaction of MgtR with MgtC. A number of small viral proteins have also been found to contain essential residues that, when mutated, eliminate protein function [16]. In contrast, another SP, the highly-conserved O3 protein of vaccinia virus, exhibits little sequence specificity requirements [17]. The O3 protein is a member of the viral entry-fusion complex. It is required for normal stability of the complex and invasion of cells by the virus. In a study designed to identify O3 amino acid sequence requirements, researchers found that while the internal transmembrane domain is essential for protein activity, no individual amino acid tested was essential for activity. Additionally, a screen for mutants with wild-type activity yielded proteins with no amino acid in agreement the wild-type protein. These studies illustrate the range of sequence requirements for different SPs.

**Fig 1:**
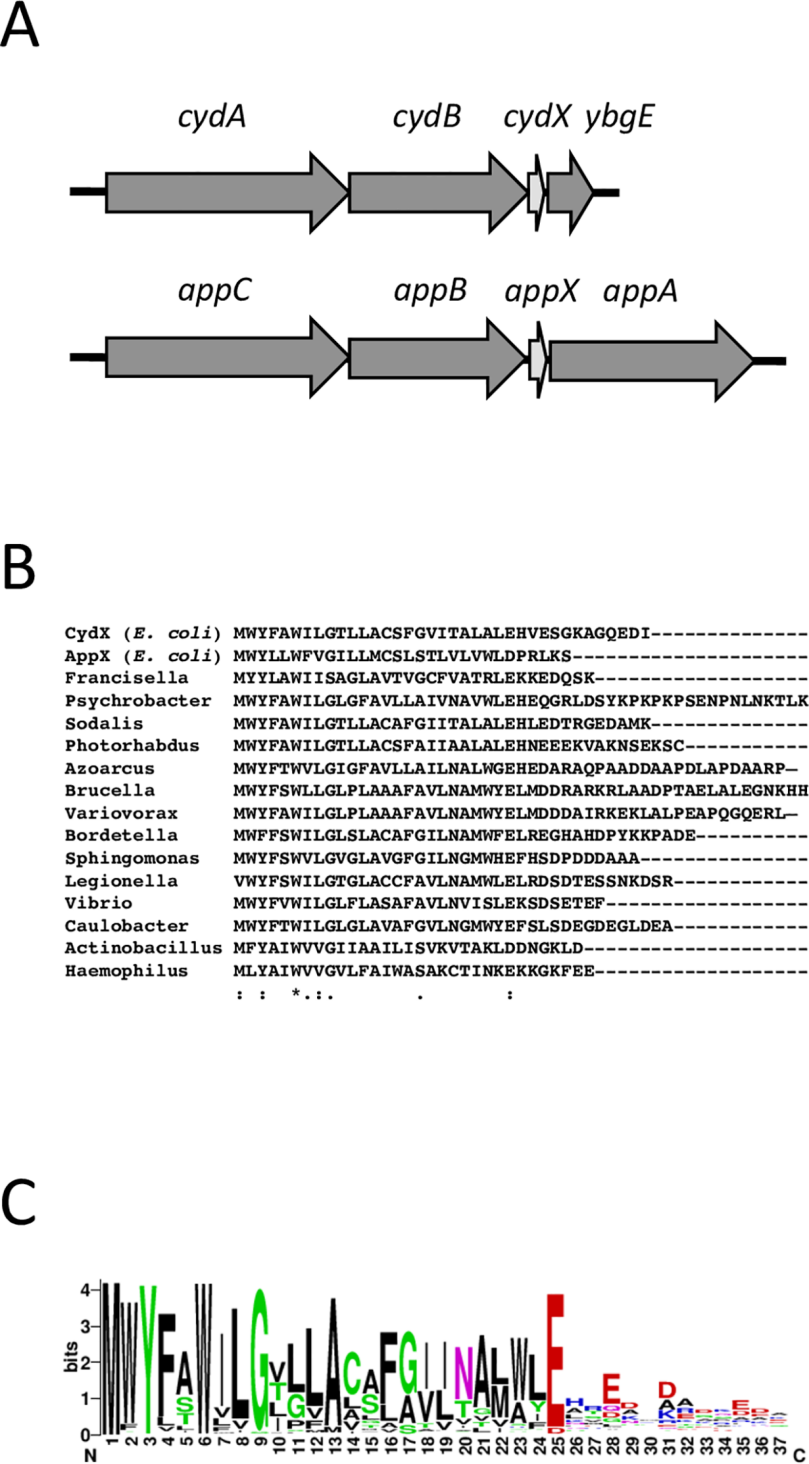
Cytochrome *bd* oxidase operons in *E*. *coli* and conservation of CydX throughout eubacteria. A) Diagram of the cytochrome *bd* oxidase operons in *E*. *coli*. B) Alignment of select CydX homologues. The alignment was generated using the program MUSCLE [24]. ‘*’ indicates that the residues are identical in all sequences, and ‘:’ and ‘.’, respectively, indicate conserved and semi-conserved substitutions as defined by MUSCLE C) WebLogo alignment of 294 CydX homologues. In the WebLogo, amino acid size correlates with degree of conservation. The sequence logo was created by the WebLogo program [25].

The small protein CydX is a subunit of the cytochrome *bd*-I oxidase complex of *E*. *coli* and other species of bacteria [10, 18–20]. Cytochrome oxidases are the terminal enzyme complexes of the electron transport chain utilized during aerobic respiration, and catalyze the reduction of molecular oxygen to water [21]. The three cytochrome *bd*-I genes, *cydA*, *cydB* and *cydX*, are encoded in a single operon in *E*. *coli* and other bacteria (Fig 1) [22,23]. CydX is conserved in over 200 species of Proteobacteria and is required for the enzymatic activity of the oxidase complex in *E*. *coli* and *Shewanella* [10, 19, 20, 23]. Deletion of the short gene results in CydABX oxidase-deficient phenotypes, such as slow growth in liquid culture, mixed colony formation, sensitivity to reductants, and reduced oxidase activity [10, 18, 20].

Although widely conserved, CydX homologues show a high degree of sequence variation. The C-terminal region varies substantially between homologues, both in sequence and length, with only a conserved set of acidic amino acids located proximal to the transmembrane helix [23]. In contrast, the N-terminal region shows more conservation, with some residues, such as Y3 and G9, being present in all but a few identified homologues. Only the tryptophan at position 6 is completely conserved in all known homologues (Fig 1). Consistent with its higher degree of conservation, characterization of the Shewanella CydX protein suggested that functionally-important residues are located at the N-terminal portion of the protein [20]. In contrast, a limited complementation study our group conducted of select CydX mutants in *E*. *coli* suggested that residues outside of the N-terminal may be essential as well [10].

The current study continues our investigation into the sequence specificity of the *E*. *coli* CydX protein. The lack of agreement between amino acid conservation and our original results were surprising, considering that less conserved residues were apparently required for complementation (I7, L12 and A21), while well-conserved residues were not required (Y3, W6, G9). Given that we were expressing these proteins at high levels from a plasmid, it seemed possible these results may be consequences of small protein overexpression [26–28] and may not reflect amino acid requirements for the endogenously-expressed protein. In addition to being artificially overexpressed, our original experimental design also did not control for other potential effects related to locus location, such as co-transcription of *cydX* with the operon and co-translational folding of CydX with CydA and CydB.

Ultimately, to more systematically evaluate the amino acid requirements of the small protein, we performed an alanine-scanning mutagenesis of the complete CydX protein in which all residues were individually mutated in *cydX* encoded at the endogenous locus in the *E*. *coli* chromosome. The resulting strains were then assayed for CydX function *in vivo*. Subsequently, select mutant alleles were reproduced with a Sequence Peptide Affinity (SPA) tag at the C-terminal end of *cydX*, which allowed for initial characterization of the mutant CydX proteins. The results of this work are consistent with previous reports that the N-terminus of CydX is important for function; however, it additionally suggests that no individual residues are essential for small protein activity. We propose that there is substantial redundancy of function between residues in CydX, which may be characteristic of multiple SPs.

## Materials and methods

### Strain construction

All strains, oligonucleotides and plasmids used in this study are listed in S1 Table, S2 Table, and S3 Table, respectively. All strains used were derivatives of the *E*. *coli* K-12 strain MG1655. CydX mutant strains were constructed by first incorporating a kanamycin cassette from the plasmid pKD4 into the genome downstream of the *cydX* gene and upstream of the *ybgE* gene (S1 Fig). Incorporation of the kanamycin gene was performed as previously described [10]. Once this *cydABX*-*kan* strain (CydX+Kan) was created, it was used as a template for PCR reactions to create all of the *cydX* mutant alleles. The *cydX*-kanamycin sequence was amplified with a mutagenic forward primer and a reverse primer that binds downstream of the antibiotic resistance cassette. These PCR products were then transformed into the recombinase-positive strain NM400. Kanamycin-resistant recombinants that contained the desired mutation in *cydX* were identified through screening by PCR using a forward primer that selectively bound to the mutated *cydX* sequence. The same process was used to make the mutated *cydX* alleles encoding the sequence peptide affinity (SPA) epitope tag at the 3’ end, except an SPA-tagged *cydX* allele was used as the starting PCR template. All strains were confirmed by sequencing. Once confirmed by sequencing, mutant alleles were transferred to a fresh MG1655 background using P1 transduction as described previously [29].

### Zone of growth inhibition assay

Assays measuring the zone of growth inhibition of different strains to β-mercaptoethanol (Sigma-Aldrich) were conducted essentially as previously described [10]. In brief, *E*. *coli* cultures were grown aerobically overnight at 30°C. The following day 200 μl of overnight cultures were added to 3 ml of top agar, vortexed, poured on LB plates containing 30 mg/L kanamycin, and allowed to solidify. A sterile disc of Whatman filter paper was placed in the center of plate and 10 μl 14 M β-mercaptoethanol (Sigma Aldrich) was applied to the center of the disk. Plates were incubated overnight at 30°C under aerobic conditions, after which the zones of growth inhibition were scored by measuring the diameter of the zone.

### Bacterial growth curves

The growth rate of wild-type and mutant strains was assayed essentially as previously described [10]. In the current experiments, cultures were grown aerobically at 37°C in LB media containing 20 mM β-mercaptoethanol. Culture growth was measured by assaying the OD_600_ of each strain every 30 minutes.

### Immunoblot assays

Immunoblots were performed to determine the relative steady-state levels of mutant CydX-SPA proteins essentially as described [29]. In brief, mutant strains were grown overnight in LB broth at 30°C. Overnight cultures were diluted 1:100, grown at 30°C, and harvested in exponential phase (OD_600nm_ of 0.4–0.6). The amount of cell extract loaded per lane was kept constant, enabling direct comparison between blots. Cell samples were lysed by boiling, and cell debris pelleted by centrifugation. Samples were separated on a 16% tricine SDS-PAGE gel and transferred to a nitrocellulose membrane. Membranes were blocked in 2% milk/PBS-T. Membranes were probed with a monoclonal anti-3xFLAG-HRP conjugated antibody in 2% milk/PBS-T (Sigma-Aldrich). Protein bands were visualized using the CDP-Star Nitro-Blocker II (Invitrogen) substrate. Membranes were then developed on film (Thermo Fisher Scientific).

### Purification of SPA-tagged CydX

Cell cultures were grown aerobically at 37°C to early stationary phase (OD_600_ 1.0–1.5) in rich media. Cell pellets were resuspended in cold purification buffer (100 mM Tris-HCl (pH 8.0), 150 mM NaCl, 10 mM EDTA, 0.2% DDM, EDTA-free protease inhibitor (Roche)) and lysed through sonication. Lysates were cleared by centrifugation and then incubated and gently rocked with anti-FLAG M2 affinity beads (Sigma-Aldrich) and buffer overnight. The lysates were passed through columns and washed twice with buffer. The final eluates were retrieved after incubation in buffer containing 0.5 mg/ ml 3X FLAG peptide (Sigma-Aldrich) diluted in purification buffer. Eluates were stored in aliquots containing an additional 10% glycerol at -80°C. Purified proteins were quantified using a dot blot dilution series. Briefly, the purified CydABX samples were serially diluted 2-fold on a dot blot and detected using an anti-FLAG antibody and chromogenic substrate. The intensity of each dot was evaluated using ImageJ software and plotted against dilution factor to determine the dilutions needed to normalize the concentrations of the purified proteins.

### TMPD oxidation assays

Equivalent quantities of purified CydABX were combined with serial dilutions of N,N,N',N'-tetramethyl-p-phenylenediamine (TMPD) and ascorbic acid at a 3:1 (TMPD:ascorbic acid) molar ratio. All solutions were diluted in purification buffer on a 96-well plate. All reactions were performed in duplicate. Oxidation of the TMPD substrate was determined by an increase in absorbance at 611 nm in a kinetic assay using a plate-reading spectrophotometer. Absorbance values were assessed once per minute for an hour. Samples without the purified CydABX were utilized as controls to subtract background oxidation. The initial velocity of each reaction was determined by graphing the absorbance over time and obtaining the slope at the beginning of the reaction. The resulting data were analyzed in GraphPad Prism (GraphPad Software) using a nonlinear fit to a Michaelis-Menten kinetic curve.

## Results

### Single amino acid alanine mutants of CydX exhibit wild-type phenotypes

**Fig 2:**
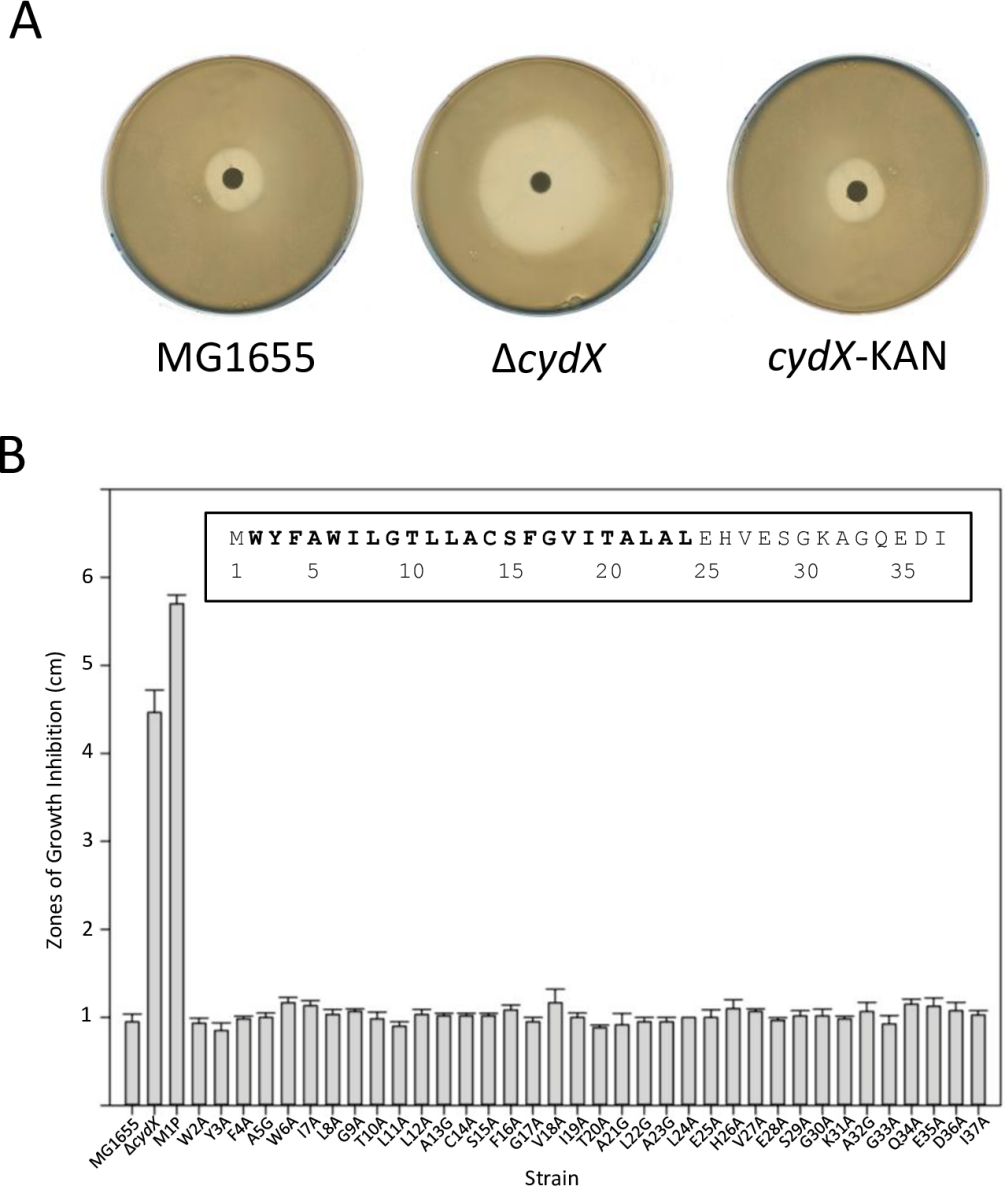
Sensitivity of single amino acid CydX mutants to β-mercaptoethanol. A) Zones of growth inhibition for wild-type, Δ*cydX* and the *cydX*+Kan template strain used to make the mutants. B) Zones of growth inhibition for each single mutant strain. The *E*. *coli* CydX protein sequence is inset. All experiments were performed in at least triplicate, and the standard error of each experiment is shown.

To identify residues that are essential for CydX function, we performed an alanine scanning mutagenesis of the *cydX* gene at its endogenous locus. The mutants were generated by inserting a kanamycin resistance cassette (KAN) downstream of the *cydX* gene in the *cydABX*-*ybgE* operon. The insertion of KAN between the *cydX* and the downstream *ybgE* gene did not lead to growth or β-mercaptoethanol sensitivity phenotypes associated with reduced oxidase activity (Fig 2). This *cydX*-*kan* strain was then used as a template for mutagenic PCR of mutant *cydX* alleles, which were then transformed into a fresh recombination-positive strain (S1 Fig). Kanamycin resistant colonies were screened for the presence of the mutation, with the success rate of mutant identification ranging widely between ~2–40% per transformation. Altogether, we were able to perform a complete alanine scanning mutagenesis of the CydX protein. Endogenous alanines were mutated to glycines, and an M1P mutant acting as a control for loss of protein.

All point mutants were assayed for phenotypes indicative of reduced cytochrome *bd* oxidase activity, including mixed colony formation, slow growth in liquid culture, and β-mercaptoethanol sensitivity. Except for the M1P mutant, which gave results consistent with being a *cydX* deletion strain, all of the single amino acid mutants displayed wild-type phenotypes (Fig 1 and data not shown). Considering the high degree of conservation of some residues in CydX, this was an unexpected result. One possible explanation of these results could be functional redundancy between CydX and AppX, a small protein paralogue encoded in the *appABX*-*appC* operon, which could allow AppX to compensate for a CydX protein with slightly-reduced activity. To test this possibility, we transduced all of the mutants into an Δ*appX* background and tested for reduced cytochrome *bd* oxidase activity in the double mutant strains. These Δ*appX*/*cydX* point mutants also exhibited wild-type β-mercaptoethanol resistance in all strains except M1P (S2 Fig). This suggests that interactions between the paralogues are not masking a mutant CydX phenotype.

### The N-terminal region of CydX is required for β-mercaptoethanol resistance

**Fig 3:**
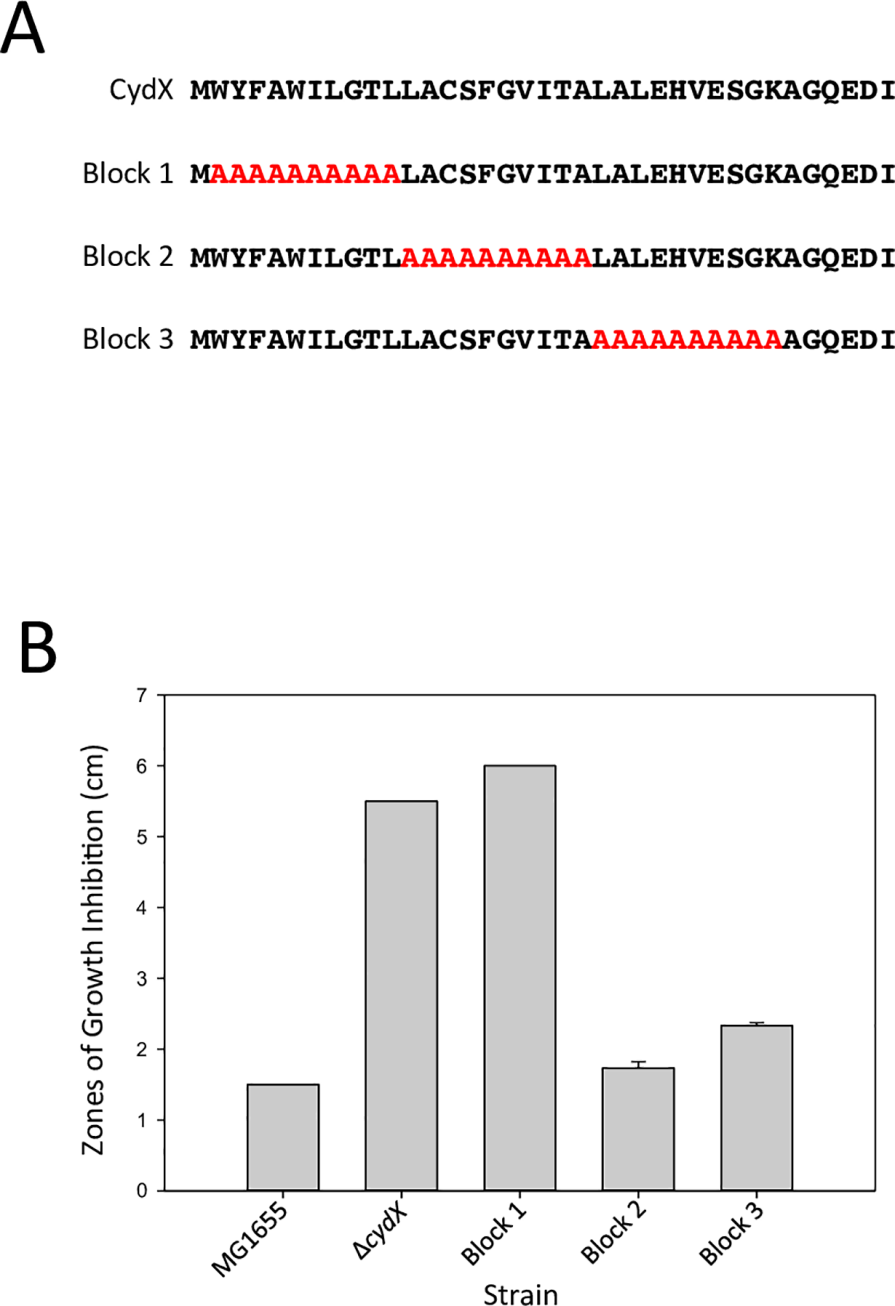
Sensitivity of alanine block CydX mutants to β-mercaptoethanol. A) Amino acid sequences of alanine block mutant CydX mutants. B) Zones of growth inhibition for wild-type, Δ*cydX*, and the three alanine block *cydX* mutants. The “Block 1” mutant contains alanines from positions 2 to 11, the “Block 2” contains alanines from positions 12–21, and the “Block 3” contains alanines from positions 22 to 31. All experiments were performed in at least triplicate, and the standard error of each experiment is shown.

Another explanation for the single mutant data is that there is a functional redundancy between residues in the CydX protein. As an initial experiment to test this possibility, a series of *cydX* mutants were created in which non-overlapping blocks of 10 consecutive alanines were substituted into the CydX protein (Fig 3). Since CydX is 37 amino acids in length, three mutants were synthesized: a substitution at residues 2–11, a substitution at residues 12–21, and a substitution at residues 22–31. A mutation of the last six residues was not constructed because previous experiments had shown that they could be deleted without an apparent effect on protein function [23]. These three mutants were tested for resistance to β-mercaptoethanol. Only one mutant, the 1^st^ alanine block mutant (residues 2–11), showed increased β-mercaptoethanol sensitivity (Fig 3), suggesting that this region of the protein is required for CydX activity and that multiple amino acids at the N-terminal of CydX may have redundant function that is lost when enough are mutated.

### CydX double and triple mutants exhibit sensitivity to β-mercaptoethanol

**Fig 4:**
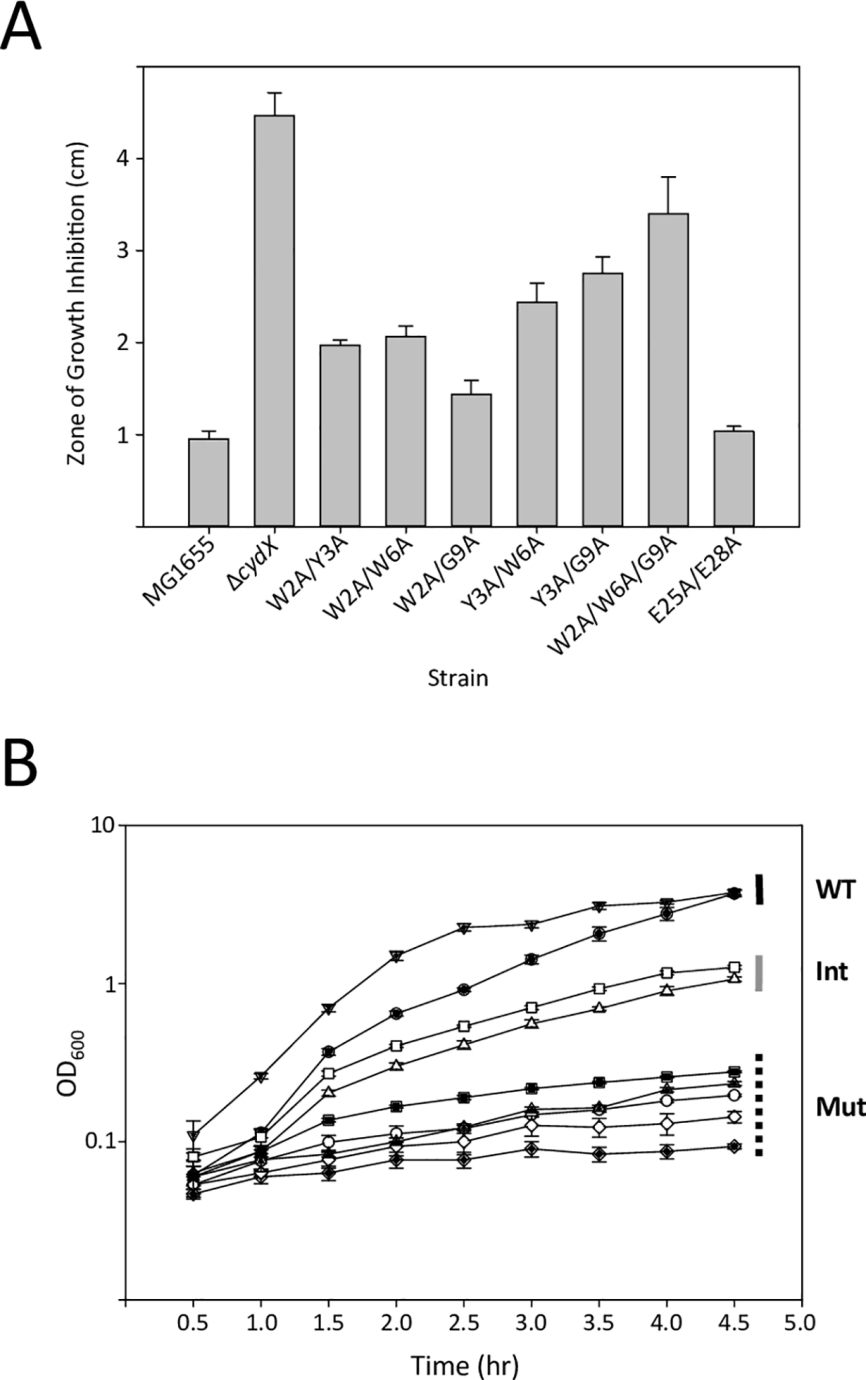
Sensitivity of double and triple amino acid CydX mutants to β-mercaptoethanol. A) Zones of growth inhibition for double and triple mutants. B) Growth of select double and triple *cydX* mutants in liquid culture containing β-mercaptoethanol. Samples are as follows: wild-type (filled circles), Δ*cydX* mutant (open circles), cydX+KAN (filled triangle), W2A/G9A mutant (open triangle), Y3A/G9A mutant (filled square), W6A/G9A mutant (open square), W2A/W6A/G9A mutant (closed diamond), Y3A/W6A/G9A mutant (open diamond), Y3A/G9A/L10G mutant (closed triangle). Strains with growth curves similar to wild-type are delineated by a dark line, strains with a growth phenotype similar to the deletion mutant are delineated with a dotted line, and strains with an intermediate growth phenotype are delineated by a grey line. Liquid culture experiments were conducted in Luria Broth containing 20mM β-mercaptoethanol. All experiments were performed in at least triplicate, and the standard error of each experiment is shown.

Although it is formally possible that all 10 amino acids in the 1^st^ alanine block mutant are required for CydX activity, it seemed likely that the phenotype is due to the loss of a select group of amino acids. Previous conservation analysis of CydX and its 259 homologues throughout the Proteobacteria showed that some residues are strongly conserved. These residues include Y3 (found in all but one homologue), W6 (completely conserved) and G9 (found in all but seven homologues) (Fig 1). Thus, these residues were the focus of our investigation to identify sets of functionally redundant amino acids. In addition, double mutants containing the W2 mutant were created to test the possibility that the two tryptophans, which are predicted to orient on the same side of the α-helix, may be redundant. A series of double mutants containing permutations of these four residues were created and tested for β-mercaptoethanol sensitivity. A double mutant of two well-conserved glutamates at the C-terminus of the protein was also tested in order to determine if eliminating these negatively charged residues, predicted to be located at the end of the hydrophobic α-helix, would impair protein function. Of those tested, the E25A/E28A double mutant showed wild-type sensitivity, but all others showed increased sensitivity relative to wild-type cells (Fig 4). The degree of sensitivity varied among the mutants, with the W2A/G9A mutant showing a moderate phenotype, while the Y3A/G9A and Y3A/W6A exhibited the most severe sensitivity. None of the double mutants, however, showed sensitivity comparable to the *cydX* deletion, suggesting that more residues may be compensating for CydX function.

To test the possibility that other amino acids were compensating in CydX function in the double mutants, two triple mutants were created to determine if a third mutation could lead to increased sensitivity to β-mercaptoethanol. The W2A/W6A/G9A and Y3A/W6A/G9A mutants showed enhanced sensitivity relative to the double mutants, and closer to the phenotype observed for the *cydX* deletion (Fig 4). Together, these data suggest that multiple amino acids at the N-terminal may be involved in CydX function, and that increased perturbation of the amino acid sequence at the N-terminal leads to reduced CydX function.

### Cell extracts enriched for CydX mutants show reduced enzymatic activity

**Fig 5:**
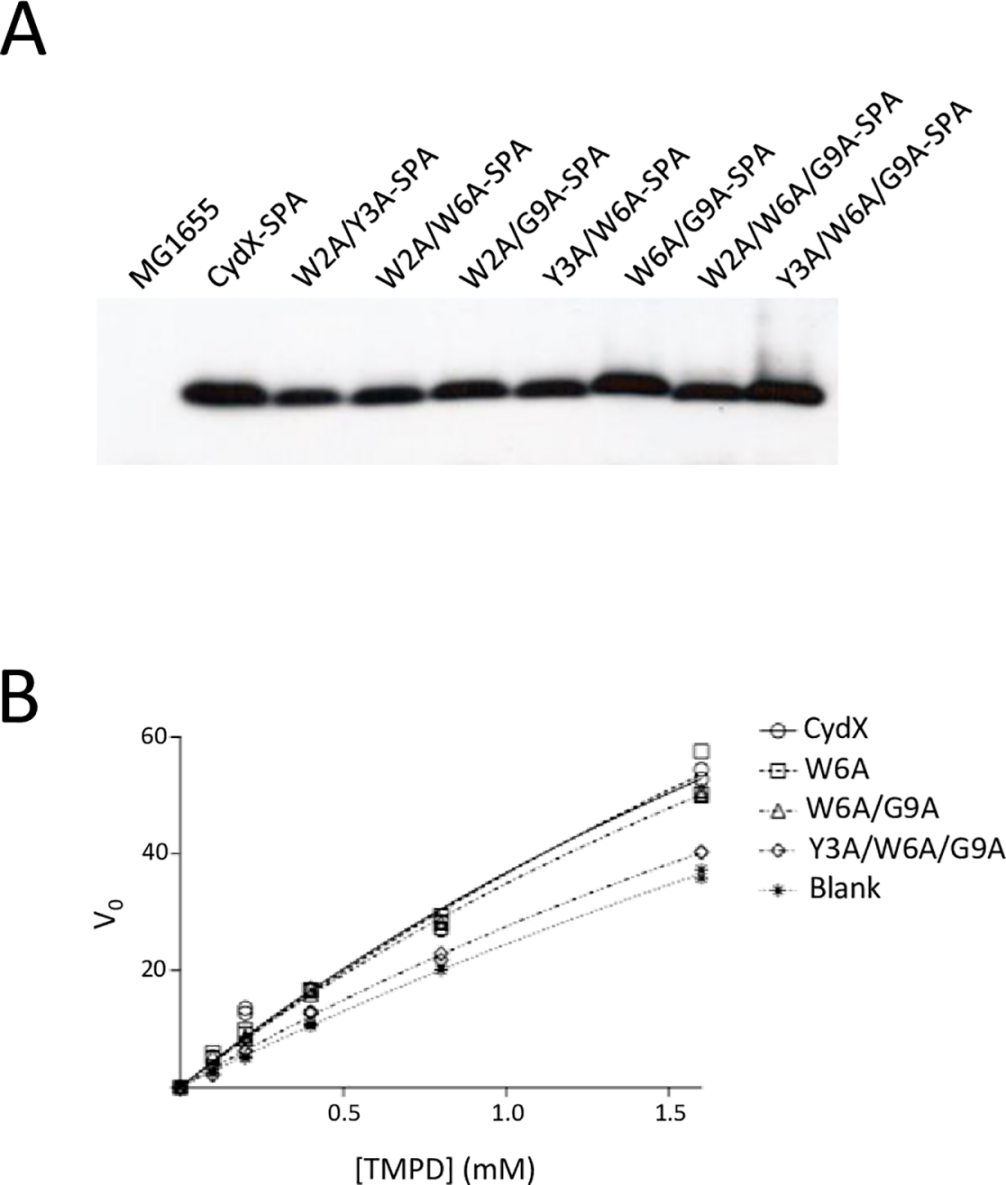
Biochemical characterization of SPA-tagged CydX mutants. A) Immunoblot analysis of steady state protein levels of SPA-tagged CydX mutant alleles. Immunoblot analysis using horseradish peroxidase-conjugated anti-3xFLAG antibodies was carried out with whole-cell extracts harvested from cultures grown to exponential phase in LB medium. B) Oxidase activity of purified extracts of SPA-tagged CydX mutant alleles. Wild-type and mutant SPA-tagged CydX proteins were purified on an anti-3xFLAG column. Oxidase activity was determined by measuring the oxidation of *N*, *N*, *N’*, *N’*-tetramethyl-*p*-phenylenediamine by the purified extracts.

In order to determine if the phenotypes observed in the double and triple mutants were due to a change in mutant protein level, the mutants were created with a C-terminal Sequence Peptide Affinity (SPA) epitope tag. Previous work has shown no effect of a C-terminal SPA tag on the function of the wild-type CydX protein [10]. Consistent with this possibility, β-mercaptoethanol zone assays of the SPA-tagged mutants yielded results similar to the untagged alleles (S3 Fig). The level of SPA-tagged double and triple mutants was then determined by immunoblot of whole cell extracts. These data showed no substantial difference in CydX protein levels between the wild-type cells and those containing mutant alleles (Fig 5). This result suggests that the phenotypes observed for the mutants is not due to an effect of the mutations on CydX abundance.

In past studies, SPA-tagged CydX purified extract showed oxidase activity toward *N*, *N*, *N’*, *N’*-tetramethyl-*p*-phenylenediamine (TMPD), a known substrate of the CydABX complex. This observation is consistent with the co-purification of the functional cytochrome *bd* oxidase complex. To determine if the *in vitro* phenotype was perturbed in mutant CydX, SPA-tagged wild-type CydX, the W6A mutant, the W6A/G9A double mutant, and the Y3A/W6A/G9A triple mutant were purified from whole cell extracts. TMPD oxidase activity was then assayed in these purified extracts. When normalized to CydX-SPA protein levels, both the single and double mutant extracts showed oxidase activity similar to wild-type, whereas the triple mutant showed little activity above that which was observed from auto-oxidation of TMPD (Fig 5). These results are consistent with the *in vivo* results showing that the single mutant has no detectable effect on small protein function, while the triple mutant has the most severe phenotype.

### Experimental inconsistencies in CydX mutational studies

In an earlier study, which was based on overexpression of mutant proteins to rescue a *cydX* deletion phenotype, a number of residues appeared to be essential for CydX function [10]. This contrasts with the current analysis using mutations at the endogenous chromosomal locus. When we observed the difference between our results, we suspected a problem with the plasmids or other reagents used in our original study. To test this, we remade plasmids that had shown only partial complementation of the *cydX* mutant, and performed all the experiments with both new and old reagents. In each case, the results of our experiments matched those previously obtained (data not shown). Thus, we are confident that our previous data are repeatable, and not the result of experimental error.

## Discussion

In this study, we mutated all 37 residues in the CydX protein using alanine-scanning mutagenesis, and assessed the functional impact of the loss of each amino acid. The high (even absolute, in the case of W6) conservation of select amino acids among diverse homologues suggested *a priori* that some residues might be essential for activity [23]. However, we were able to mutate each residue without observing phenotypes associated with a loss of CydX function. A broader mutagenesis of 10 amino acid blocks did affect activity, but only when located at the N-terminal region of the protein. These results are consistent with previous work demonstrating that the protein’s N-terminus is important for activity [20], and suggest that this region is a site of functionally-important interactions with other proteins in the complex. The data also demonstrate that CydX function is not substantially affected by single point mutations or 10-residue substitutions in the predicted transmembrane region or the region directly C-terminal to the transmembrane region. Together, these results suggest a very high degree of CydX sequence plasticity.

A number of studies have shown that hydrophobic regions in proteins will retain function even after substantial mutation [30–32]. Core residues of bacteriophage T4 lysozyme, the ribonuclease barnase, and thioredoxin all show sequence plasticity at hydrophobic α-helices involved in maintaining protein structure [31,32]. Similar to these core hydrophobic regions, there is evidence that CydX may function to increase stability of the oxidase complex [18,19]. If the primary role of CydX is to maintain protein complex stability, the observed sequence plasticity is consistent with that observed in other protein regions with similar function. However, the strong, and even absolute, conservation of individual residues at the N-terminus suggests that selective pressure is maintained on these residues. One potential reconciliation of these data is the possibility that mutations at these highly-conserved residues lead to other functional consequences beyond those studied here. It will be interesting to see if, upon further characterization of CydX and other small proteins, the reason for selective pressure on these residues becomes apparent.

In contrast to the single mutants, increasing the mutational load at the N-terminus with double and triple mutants leads to decreased cytochrome *bd* oxidase function. This suggests that interaction of CydX with the complex may not be dependent on individual amino acids, but may instead be coordinated through a combination of interactions between multiple residues. A similar conclusion was reached in a study of the O3 protein from vaccinia virus [16]. Like CydX, the O3 protein is highly conserved and essential for normal organism function, but shows significant sequence variability among homologues. Also similar to CydX, no individual residues were found to be essential for O3 function. This raises the intriguing question of interaction-specificity between CydX or O3 and their respective partners. The potential plasticity in hydrophobic small protein interactions with protein partners is also illustrated in a recent study showing that synthetic small proteins containing only leucines and isoleucines can interact specifically with, and also activate, the platelet-derived growth factor β-receptor [33]. Together, these results support the idea that for some hydrophobic small proteins, interactions with protein partners may be determined by a sequence-flexible overall structure, rather than requiring a few essential residues.

This study continues a preliminary analysis we conducted of CydX amino acid specificity requirements [10]. In our earlier study, we tested for complementation of the *cydX* deletion mutant using wild-type and mutant *cydX* alleles expressed *in trans* from a high-copy number plasmid. We designed the current study to test the effect of *cydX* mutations when expressed from the endogenous locus within the cytochrome *bd* oxidase operon. In our current experiments, it is more likely that the mutant proteins will be expressed, synthesized and integrated into the CydABX complex similarly to the wild-type protein. The fact that we were able to replicate our previous results with new plasmids and reagents suggests that our previous results were not due to experimental error, but rather experimental design. Ultimately, this may be a cautionary example of using overexpressed proteins expressed *in trans* to test for SP functionality.

## Supporting information

S1 Fig Diagram of *cydX* mutant allele construction on the *E*. *coli* chromosome. (PDF)

S2 Fig Sensitivity of single amino acid CydX mutants in a Δ*appBCX* background to β-mercaptoethanol. (PDF)

S3 Fig Sensitivity of SPA-tagged CydX mutants. (PDF)

S1 Table Strains in this study. (XLSX)

S2 Table Oligomers used in this study. (XLSX)

S3 Table Plasmids used in this study. (XLSX)
