# Supplementary material for: Investigation of amino acid specificity in the CydX small protein shows sequence plasticity at the functional level
Source: PLoS One. 2018 Jun 18;13(6):e0198699. doi: 10.1371/journal.pone.0198699 (PMC6005532; doi:10.1371/journal.pone.0198699)
Supplement: S1 Fig — (PDF) [file pone.0198699.s001.pdf]

A

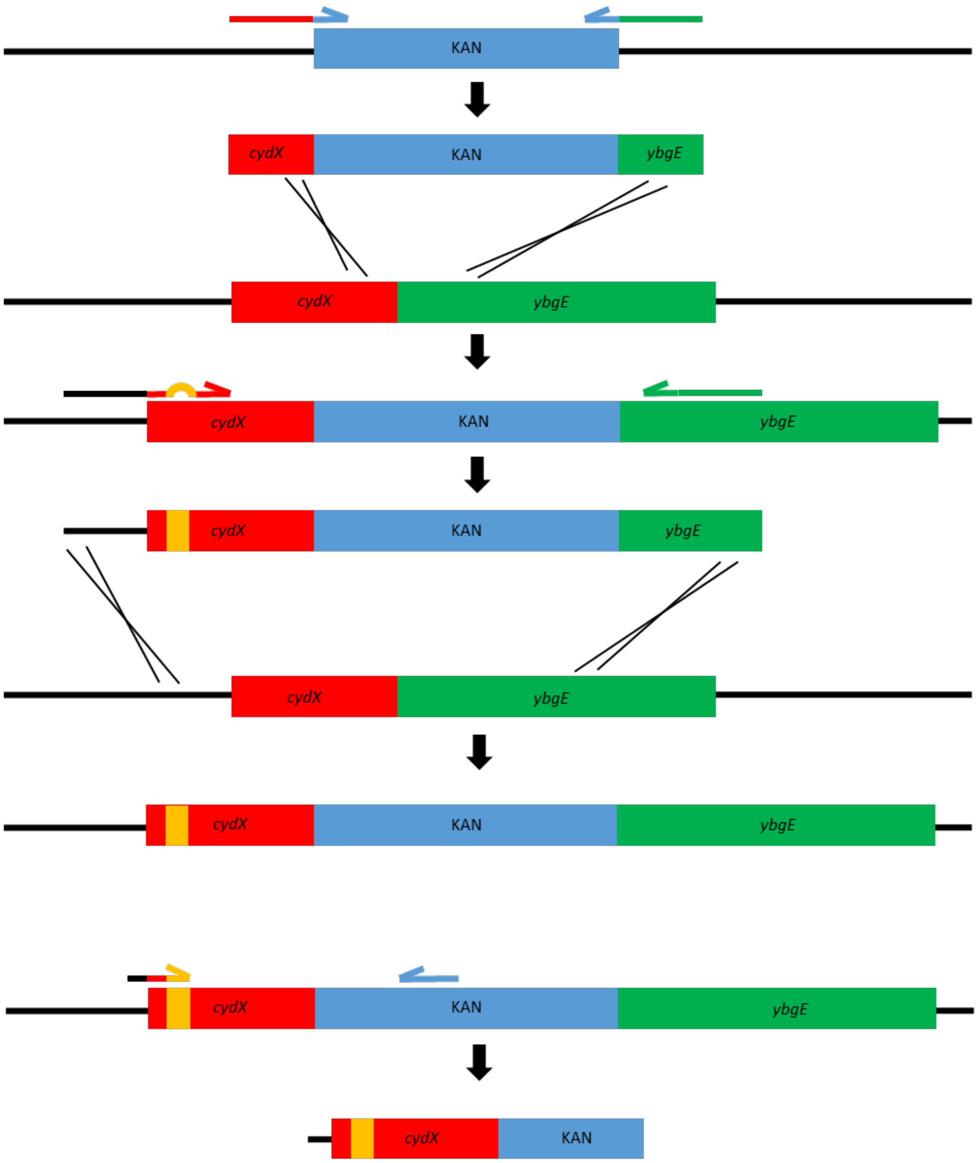
KAN
cydX
KAN
ybgE
cydX
ybgE
cydX
KAN
ybgE
cydX
KAN
ybgE
cydX
ybgE
cydX
KAN
ybgE
cydX
KAN
ybgE
cydX
KAN

B

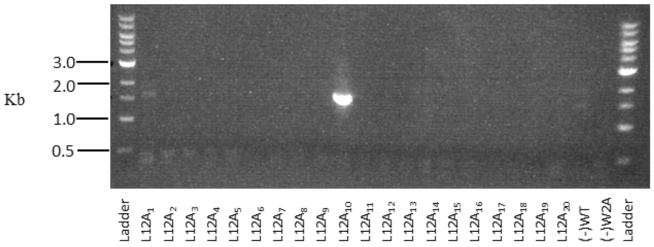
Kb
3.0
2.0
1.0
0.5
Ladder
L12A1
L12A2
L12A3
L12A4
L12A5
L12A6
L12A7
L12A8
L12A9
L12A10
L12A11
L12A12
L12A13
L12A14
L12A15
L12A16
L12A17
L12A18
L12A19
L12A20
(-)WT
(-)W2A
Ladder

S1 Fig

Supplemental File 1. Construction of the *cydX* mutant alleles at the endogenous locus. (A) Diagram showing the mutant synthesis strategy as outlined in the Materials and Methods. (B) Example results from screening Kan(R) NM400 transformants for those where recombination had taken place outside of the desired mutation.

S1 Fig
