# Supplementary figures and images for: Investigation of amino acid specificity in the CydX small protein shows sequence plasticity at the functional level
Source: PLoS One. 2018 Jun 18;13(6):e0198699. doi: 10.1371/journal.pone.0198699 (PMC6005532; doi:10.1371/journal.pone.0198699)

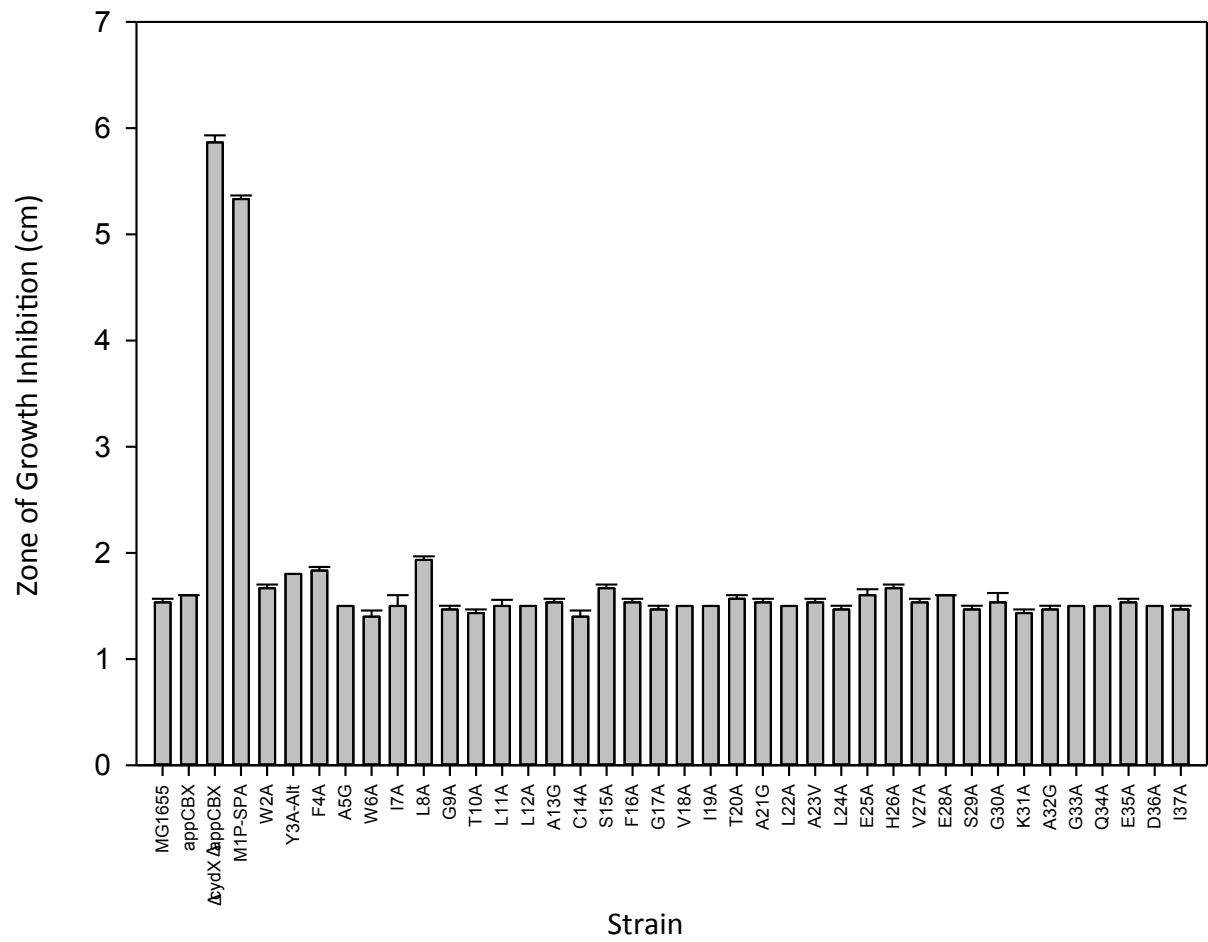

S2 Fig

Supplement: S2 Fig — (PDF) [file pone.0198699.s002.pdf]

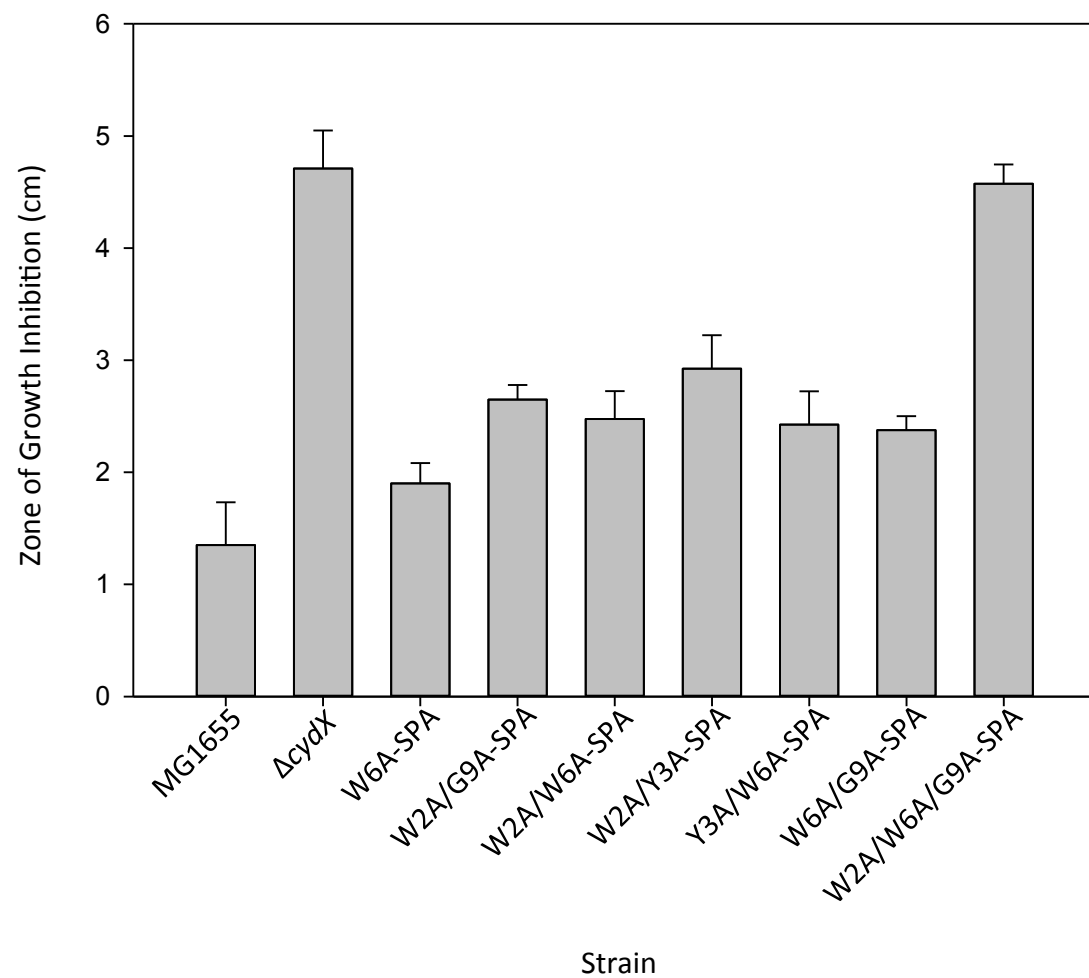

S3 Fig

Supplement: S3 Fig — (PDF) [file pone.0198699.s003.pdf]
